# Artificial Intelligence-Based Video Analysis for Assessing Sucking Behavior in Preterm Infants: A Feasibility Study

**DOI:** 10.3390/children13040479

**Published:** 2026-03-30

**Authors:** Ji Ae Kim, Jihye Chae, Su Min Kim, Eui Kyun Lee, Seung Hak Lee, Seungwoo Cha, Garam Hong, Jihoon Kweon, Eun Jae Ko

**Affiliations:** 1 Department of Physical Medicine and Rehabilitation, Korea University Guro Hospital, Seoul 08308, Republic of Korea; 2 Department of Rehabilitation Medicine, Asan Medical Center, University of Ulsan College of Medicine, Seoul 05505, Republic of Koreaeamsmed@gmail.com (G.H.); 3 Department of Convergence Medicine, Asan Medical Center, Seoul 05055, Republic of Korea; 4 Department of Rehabilitation Medicine, Asan Medical Center, Seoul 05055, Republic of Korea

**Keywords:** artificial intelligence, infants, preterm, swallowing

## Abstract

**Highlights:**

**What are the main findings?**

- An AI-based video analysis framework using facial keypoint tracking achieved an overall classification accuracy of 82.76% for Normal and Disorganization and 96.55% for Dysfunction in preterm infant feeding sessions compared to NOMAS expert evaluation.

**What is the implication of the main finding?**

- AI-based video analysis of bottle-feeding sessions offers a feasible, noninvasive alternative to conventional dysphagia screening tools, enabling objective assessment by non-specialists in NICU settings without radiation exposure.

**Abstract:**

Background/Objectives: Preterm infants often experience impaired swallowing function, and objective assessments for this population remain limited. In this prospective single-center study, we aimed to propose and validate an automated framework that quantitatively assesses neonatal sucking behavior by tracking facial key points in bottle feeding videos. Methods: Fifty-eight preterm infants (corrected age [CA] ≤ 2 months) were enrolled, and 2 min videos of bottle-feeding were recorded. Certified therapists manually evaluated the videos using the Neonatal Oral Motor Assessment Scale (NOMAS), and an artificial intelligence (AI)-based analysis classified the videos into the following three groups: Normal, Disorganization, and Dysfunction. At 12 months CA, developmental outcomes were assessed using the Mental Development Index (MDI) and the Psychomotor Development Index (PDI) of the Bayley Scales of Infant Development, Second Edition (BSID-II). Results: Among the 58 infants, the AI-based tool correctly classified 47 and misclassified 11. The classification accuracy was 82.76 for the Normal group, 82.76 for Disorganization, and 96.55 for Dysfunction. The mean PDI was lower in the Dysfunction group than in other groups; however, the differences were not statistically significant. Conclusions: This novel AI-based video analysis demonstrates preliminary potential as a noninvasive tool for evaluating sucking behavior in preterm infants, potentially enabling early identification of dysphagia even by non-specialists in the neonatal intensive care unit (NICU) without hazard exposure. This feasibility study demonstrates preliminary technical viability of a video-based framework for neonatal sucking behavior assessment; however, further validation is required before clinical implementation.

## 1. Introduction

Although 13.5 million newborn babies were born preterm in 2020, advancements in neonatal intensive care have significantly improved the survival rates of premature infants born before 37 weeks of gestation [1,2]. Oral feeding is a crucial developmental milestone essential for appropriate growth and neurodevelopment [3,4]. However, achieving this milestone is particularly difficult for preterm infants. Failure to do so may result in poor weight gain and developmental delays [5,6,7]. The prevalence of dysphagia (difficulty in swallowing) among preterm infants is rising [8]; for instance, Dewi et al. recently reported that >20% of preterm infants are affected [9]. Therefore, early detection and timely intervention are essential to support the development of normal swallowing function and help preterm infants reach key developmental milestones. The videofluoroscopic swallowing study (VFSS) is the diagnostic tool used for evaluating dysphagia in infants; however, VFSS presents significant challenges in clinical settings, including radiation exposure, which limits its routine use [10].

The Neonatal Oral Motor Assessment Scale (NOMAS), which assesses swallowing function in infants of up to 48 weeks of postmenstrual age through observation during feeding, is often used as a screening tool for dysphagia in place of videofluoroscopy [11,12]. NOMAS has been rigorously evaluated for its psychometric properties and remains the only instrument suitable for assessing both breastfed and bottle-fed newborns and infants [4,13,14]. However, one notable limitation of its psychometric performance is the low inter-rater reliability observed in preterm infants who exhibit disorganized sucking patterns [15,16]. Revisions have been introduced to improve reliability [17], and NOMAS has a major advantage in that it involves no radiation exposure. Therefore, in the neonatal intensive care unit (NICU) setting, it is considered one of the best available clinical screening tools for evaluating swallowing function [18].

Automated image-based artificial intelligence (AI) analyses offer a promising opportunity to develop objective and consistent tools that address the reproducibility issues associated with subjective visual ratings of NOMAS. Recent advances in computer vision have demonstrated that video-based AI systems can match or even exceed human performance in neonatal assessments. For example, an automated classifier [19] analyzing overnight crib videos achieved precision and recall rates > 80% in distinguishing infant sleep–wake states [20]. Similarly, AI-driven facial expression analysis has shown strong correlations with expert clinicians’ pain assessments (r = 0.84–0.86) during neonatal procedures. Video-based methods are also being explored to analyze feeding behavior. Notably, a recent study using standard baby monitor footage reported 96% accuracy in detecting non-nutritive sucking episodes [21].

In this study, we aimed to propose a dysphagia screening tool for preterm infants with an automated framework that quantitatively assesses neonatal sucking behavior by tracking facial key points in bottle feeding videos. To achieve stable long-term tracking, we integrated a geometry-aware module with a CoTracker2 to mitigate drift in tracking points. Based on the sucking patterns identified from the tracking data, we developed a classification logic for feeding sessions in accordance with the NOMAS criteria [22]. The accuracy of AI-based classification was evaluated in comparison with NOMAS assessments performed by certified therapists, with the ultimate goal of establishing this framework as a feasible and objective screening tool for dysphagia in preterm infants.

## 2. Materials and Methods

This study was approved by the Institutional Review Board (IRB) of the Asan Medical Center (IRB No. 2022-0994), and was also registered with the Clinical Research Information Service (registration number: KCT0007607), and all methods were performed in accordance with relevant guidelines and regulations. All video recordings were stored on a password-protected, institutional server accessible only to the research team. Parental informed consent explicitly covered the recording, storage, and computerized analysis of facial video data for research purposes, in accordance with the IRB-approved protocol.

### 2.1. Participants

In this prospective single-center study, preterm infants who visited the outpatient clinic of the Department of Pediatric Rehabilitation Medicine at Asan Medical Center between July 2022 and October 2024 were assessed for inclusion according to the following criteria: (1) preterm infants born before 37 weeks of gestation; (2) children younger than 2 months of corrected age at the time of enrollment, and (3) children whose parents provided written informed consent. Exclusion criteria were as follows: (1) infants who were breastfeeding, and (2) infants unable to feed orally at the time of enrollment.

### 2.2. Video Recording

**Figure 1:**
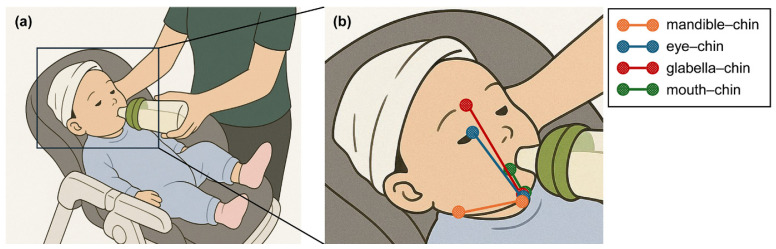
(**a**) Setup for video recording during feeding sessions at a 45° angle relative to the sagittal plane. (**b**) Data labeling on facial key points for tracking.

A video was recorded for each infant during a feeding session, defined as a 2 min bottle-feeding period. A video was recorded during each feeding session at a 45° angle relative to the infant’s sagittal plane by trained clinical staff (Figure 1a). The recording captured the infant’s full body while seated in a cradle, using mobile phones (Galaxy Z flip3 5G, Android 11, Samsung, Seoul, Republic of Korea). A frame rate of 30 fps and resolution of 1080 × 1920 pixels was obtained for the recordings.

### 2.3. NOMAS

Feeding videos were manually evaluated by two therapists with NOMAS certification to establish the ground truth. NOMAS consists of a 28-item checklist that assesses feeding patterns [23]. Two NOMAS-certified therapists independently evaluated all feeding videos and classified them as Normal, Disorganization, or Dysfunction [18]. To assess inter-rater reliability, Cohen’s kappa was calculated between the two raters, yielding a moderate level of agreement (κ = 0.598). Cases with discordant ratings were reviewed and resolved through discussion to reach a final consensus. This approach aimed to reduce the influence of individual bias and enhance the reliability of the manual classification used as the reference. According to NOMAS criteria, a “Normal” feeding session is characterized by successful coordination of the infant’s suck–swallow–breathe responses and efficient feeding. Infants who exhibit difficulty with this coordination are classified as having “Disorganization,” while abnormal oral motor movements that interfere with feeding result in a classification of “Dysfunction.” Characteristics of Dysfunction include excessive jaw excursions that compromise the nipple seal, lateral jaw deviation, a flaccid or retracted tongue, or complete absence of movement [15,18].

### 2.4. Data Labeling

**Figure 2:**
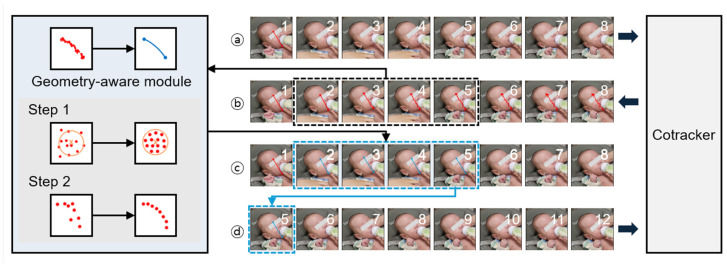
Geometry-aware tracking pipeline. (**a**) The raw video is divided into eight-frame sliding windows, with manual key point annotation performed only on the first frame of the entire sequence. (**b**) CoTracker then propagates these key points to the subsequent frames within the current window (frames 2–8). (**c**) Predicted coordinates are corrected using constraints specific to the label’s geometric type (line or circle). (**d**) The last corrected frame (frame 5) serves as the labeled anchor for the subsequent window, repeating the track–refine–propagate cycle until the video concludes. The authors affirm that written informed consent was obtained from all human participants for the publication of any potentially identifying images.

To reduce the computational burden of tracking, each video was cropped to include only the infant’s facial region. The first frame of each video was then manually annotated with labels. Each label set comprised a dumbbell-shaped cluster of points: two circles placed on facial key points and the line connecting them. The key point pairs examined—mandible–chin, eye–chin, glabella–chin, and mouth–chin (Figure 2b)—represent critical anatomical landmarks for swallowing function analysis and NOMAS assessment. Labels were generated and systematically managed using the Computer Vision Annotation Tool.

### 2.5. Label Tracking

**Figure 3:**
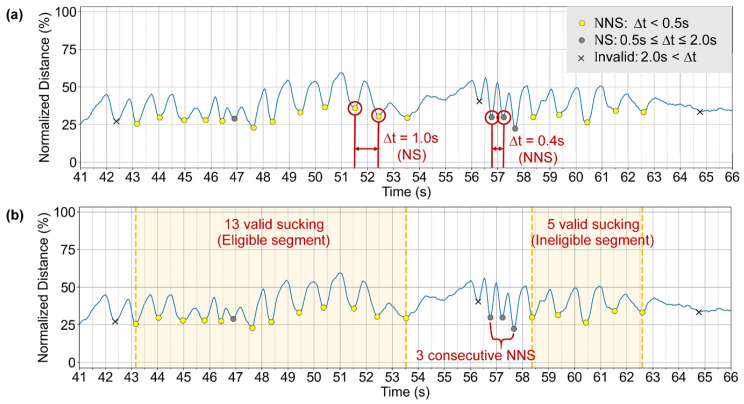
Diagnostic algorithm based on a three-step hierarchical process for feeding session classification. (**a**) Peak validation example. Each detected peak is then categorized by the time elapsed (Δt) since the previous one: a Non-Nutritive Suck (NNS; gray dot) for Δt < 0.5 s, a Nutritive Suck (NS; yellow dot) for 0.5 ≤ Δt ≤ 2.0 s, or an Invalid peak (black cross) for Δt > 2.0 s. (**b**) Segment validation example: The first segment is eligible with 13 valid peaks. It is followed by three consecutive NNSs, which are invalidated, thus terminating the segment. The next segment begins thereafter but is deemed ‘ineligible’ as it contains only five valid peaks.

A geometry-aware module was integrated with CoTracker2 to prevent drift of the tracking points (Figure 3) [22]. CoTracker2 performed tracking using sliding windows of eight consecutive frames, taking the labeled first frame of each window as input and outputting the tracked coordinates for the remaining seven frames. The coordinates associated with each key point were then refined using a radius-based correction constrained by a convex hull. Next, the curve connecting every key point pair was adjusted to preserve their structural relationships using a piecewise cubic Hermite interpolating polynomial. The corrected coordinates were subsequently used as the label for the first frame of the next window.

### 2.6. Parameters

To characterize sucking patterns, we considered the following two variables: (1) the Euclidean distance between two facial key points and (2) the angle between the line connecting those key points and the x-axis. Tracking examples for the four key point pairs are provided in Supplementary Figure S1. Upper and lower peaks were automatically identified in the min–max normalized variables using a local peak detection algorithm [24]. Peak detection parameters were tuned to minimize false predictions. To eliminate minor fluctuations irrelevant to sucking movements, a minimum peak interval was imposed to suppress spurious peaks, and constraints were applied to peak-to-peak duration to account for inter-individual variability (Supplementary video).

### 2.7. Tracking-Based Categorization of Feeding Sessions

**Figure 4:**
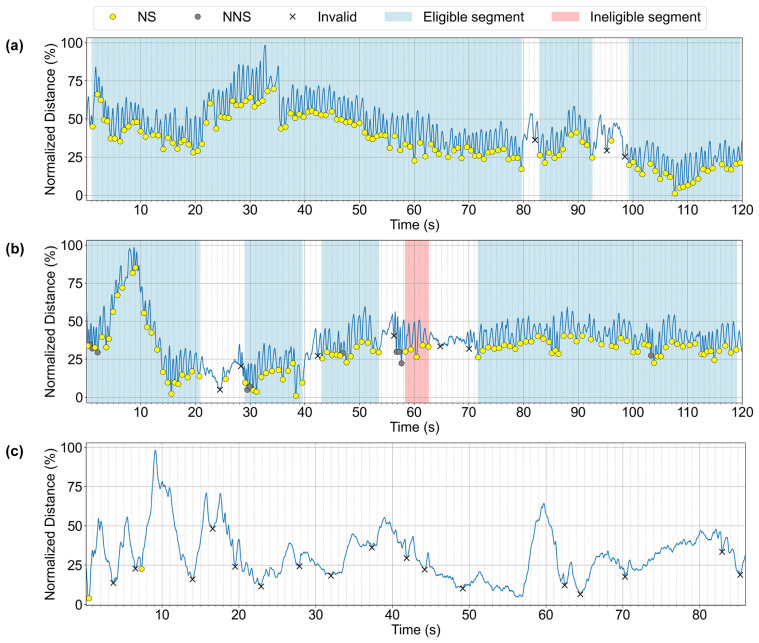
Representative examples for AI-based categorization of feeding sessions into (**a**) Normal, (**b**) Disorganization, and (**c**) Dysfunction.

The diagnostic categorization logic applied in this study is rule-based and deterministic, with thresholds defined a prior in accordance with NOMAS clinical criteria. Based on the NOMAS criteria, we developed a diagnostic algorithm (Figure 4) to classify feeding sessions. The algorithm follows a three-step hierarchical process: (1) assessing the validity of individual sucking events, (2) identifying segments in which valid sucking occurs consecutively, and (3) evaluating the overall feeding session based on the number of valid sucking events within each segment. Among the four tracked label pairs, the glabella–chin pair was selected as the most representative of the infant’s swallowing pattern. Temporal variations in the distance between the glabella and chin were analyzed as input for the diagnostic algorithm.

In the sucking assessment, a single sucking event was identified using the lower peak of the distance between the glabella and chin, which corresponds to the moment of mandibular elevation (Figure 4a). The first peak in a feeding session was initially considered valid. Each subsequent peak was considered valid if the interval from the preceding peak was ≤2 s. Valid peaks were further classified as non-nutritive sucking (NNS) (inter-peak interval < 0.5 s) or nutritive sucking (NS) (0.5 s ≤ inter-peak interval ≤ 2 s).

A segment was defined as a sequence of at least three consecutive valid peaks, beginning with an NS event. A segment containing ≥10 valid peaks was considered eligible, whereas one with <10 valid peaks was labeled ineligible. Segments were divided at occurrences of three or more consecutive NNS events, and the NNS sequence itself was excluded from the resulting segments. To avoid underestimation of sucking counts due to video boundary effects, any segment that ended within the first 5 s of the recording or began within the last 5 s was excluded from evaluation.

**Figure 5:**
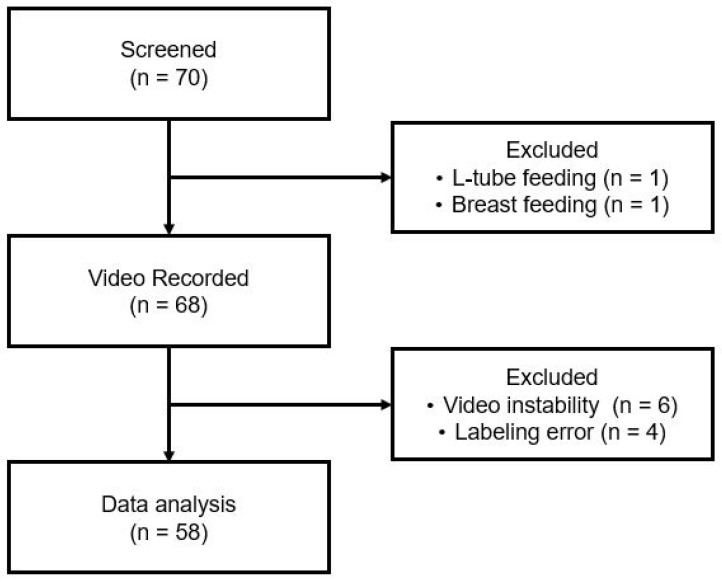
Patient enrollment criteria.

Feeding sessions composed entirely of eligible segments were classified as ‘Normal’ (Figure 5). Sessions that included a mixture of eligible and ineligible segments were labeled ‘Disorganization,’ whereas sessions in which no eligible segment was detected were categorized as ‘Dysfunction.’

### 2.8. Long-Term Development Follow up with Bayley Scales of Infant Development–II

At a corrected age of 12 months, the Bayley Scales of Infant Development–II (BSID-II) were administered by two experienced occupational therapists to assess the overall developmental status of the participants. Two domains were evaluated, the mental developmental index (MDI) and the psychomotor developmental index (PDI), which assess cognitive and motor development, respectively [25,26]. The MDI assesses abilities such as problem-solving, imitation, memory, and hand-eye coordination, while the PDI evaluates gross and fine motor skills [27]. Based on standardized scoring criteria, MDI and PDI scores are classified into four categories: accelerated (score: ≥115; >+1 SD), within normal limits (scores: 85–114), mildly delayed (scores: 70–84; −1 SD), and significantly delayed (score: ≤69; −2 SD) [28].

### 2.9. Other Measurements

The clinical information collected included gestational age at birth, birth weight, corrected age at the time of participation, sex, and the presence of brain lesions.

### 2.10. Statistical Analysis

Descriptive statistics were used to summarize baseline characteristics. Sensitivity and specificity were calculated for each measurement using the one-vs-rest (OvR) strategy, which transforms a multi-class problem into multiple binary classification tasks. The OvR approach involves selecting one class as the “positive” class and combining all other classes into a single “negative” class [29]. Kruskal–Wallis analysis was performed to compare PDI, MDI, and chronological age across the Normal, Disorganization, and Dysfunction groups. All statistical analyses were conducted using SPSS version 25 (IBM Co., Armonk, NY, USA). Statistical significance was set at *p* < 0.05.

## 3. Results

Among the 70 preterm infants screened, infants who were exclusively breastfed (*n* = 1) and those with nasogastric (L-tube) feeding who could not feed orally (*n* = 1) were excluded. Furthermore, videos were excluded from analysis when artifacts caused by the caregiver rocking the infant to encourage sucking were present (*n* = 6), or when facial occlusion by the infant’s arm occurred within the first 60 s (*n* = 4). Ultimately, 58 infants were included in the AI-based analysis (Figure 1).

Among the 58 preterm infants included in the study (Table 1), 43.1% were born between 28 and 32 weeks of gestational age. The mean birth weight was 1189 ± 519 g, and the mean corrected age at the time of video analysis was 5.1 ± 2.3 weeks. A total of 33 infants (56.8%) had a history of brain injury. The mean MDI score of the BSID-I was 86.0 ± 13.7, and the mean PDI of the BSID-II was 78.1 ± 16.0 at the corrected age of 12 months.

### 3.1. AI-Based Categorization Compared to the Manual Analysis of NOMAS

**Figure 6:**
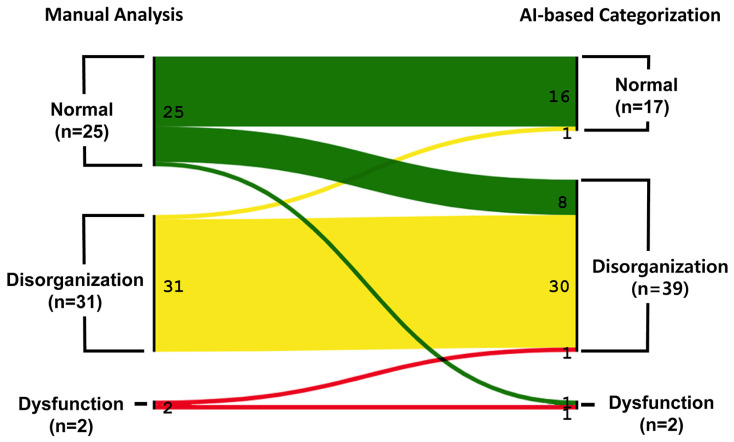
Sankey diagram illustrating the transition from manual analysis to AI-based classification of feeding sessions. N 16. N > DO = 8; N > DF = 1; DO > N = 1; DO > DO = 30; DO > DF = 0; DF > N = 0; DF > DO = 1; DF > DF = 1.

In the manual assessment, among the 58 preterm infants, 25 were classified as Normal, 31 as Disorganization, and 2 as Dysfunction (Figure 6). Of these, 47 infants were correctly classified by the AI-based analysis (Table 2). Most misclassifications involved Normal infants being predicted as Disorganization (*n* = 8), while only one Disorganization case was misclassified as Normal (1 out of 31). Consequently, the AI-based analysis achieved a high sensitivity of 96.77% for detecting Disorganization. The micro-average score was 0.81, the macro-average F1 score was 0.71, and the weighted-average F1 score was 0.80.

### 3.2. Neurodevelopmental Outcomes by NOMAS Classification

**Figure 7:**
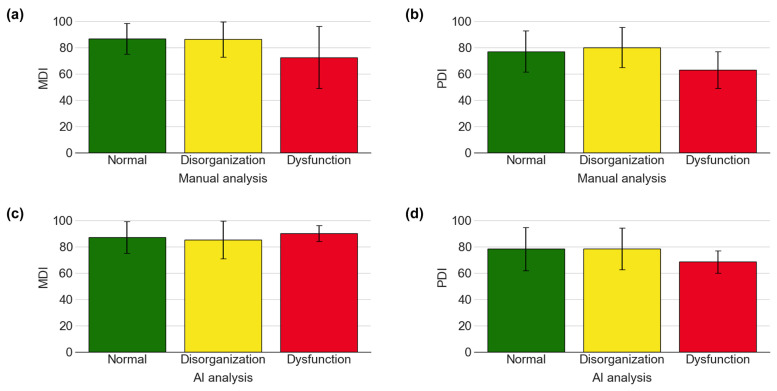
(**a**) MDI of BSID-II in three groups categorized by manual analysis, (**b**) PDI of BSID-II in three groups categorized by manual analysis, (**c**) MDI of BSID-II in three groups categorized by AI analysis, (**d**) PDI of BSID-II in three groups categorized by AI analysis.

In this study, we investigated the relationship between the categorization of feeding videos (Normal, Disorganization, and Dysfunction) and developmental outcomes, as measured by the BSID-II, in 52 infants. No statistically significant differences were found in the MDI or PDI across the three categories in either the manual or AI-based analyses (Table 3, Figure 7).

## 4. Discussion

In this study, we developed a novel AI-based approach for analyzing bottle-feeding sessions in preterm infants. To enable continuous tracking of facial points, we integrated a geometry-aware module into Cotracker2. Furthermore, we implemented a three-step hierarchical process—sucking, segment, and patient classification—in alignment with the NOMAS criteria. Despite a few misclassified cases, our approach demonstrated the feasibility of an objective and scalable diagnostic tool for early feeding assessment. Unlike the conventional NOMAS assessment [16,30], which relies on subjective bedside observations, our AI method applies consistent analytical criteria to video recordings. This has the potential to reduce inter-rater variability and enable at-home monitoring by non-specialists, such as parents, thereby supporting earlier identification of feeding difficulties and timely clinical intervention.

Among the 58 infants evaluated, the AI-based analysis accurately classified most feeding sessions, with 11 cases identified as misclassifications. The most common error involved Normal cases being incorrectly categorized as Disorganization, primarily due to the failure to detect valid sucking behaviors. Similarly, inadequate key point tracking throughout the feeding session resulted in an absence of valid peaks, which led to the misclassification of a Normal case as Dysfunction. One case of Disorganization was misclassified as Normal because the AI-based analysis did not include logic to identify short inter-burst pauses less than 2 s—used by therapists to detect disrupted rhythmic jaw movement during manual assessment. A case of Dysfunction was also classified as Disorganization, as the AI-based analysis lacked the ability to evaluate structural or morphological features of the mouth. In the manual analysis using the NOMAS criteria, Dysfunction is diagnosed when clinical signs—such as incomplete nipple–oral seal or minimal jaw excursion caused by flaccid tone or spasticity—are present, which cannot yet be evaluated by AI-based methods. These findings suggest that while the AI-based system performs well in identifying Normal and Disorganized sucking patterns, further refinement may be needed to improve sensitivity in detecting Dysfunction. At this stage of development, a binary Normal vs. Abnormal framing may be more clinically appropriate and statistically tractable for future studies.

When comparing our approach with existing assessments of swallowing function, several key distinctions emerge. Traditional methods, such as VFSS and fiberoptic endoscopic evaluation of swallowing (FEES), provide direct visualization of swallowing physiology but are invasive and require specialized equipment [31,32]. In contrast, our AI-based analysis offers a noninvasive alternative that can be implemented in routine clinical settings.

In a previous study, Sazonov et al. assessed nutritive sucking patterns using an instrumented feeding bottle equipped with a pressure sensor, which provided moderately accurate information on suck count [33]. While this method offered quantifiable data on basic sucking metrics, our approach goes beyond simply counting sucks. It provides sucking frequency to distinguish between NS and NNS, and it evaluates both the quantity and quality of sucking. These features enable automated categorization of overall feeding patterns according to the NOMAS criteria. Our video analysis method may preserve this critical clinical distinction, which guides treatment decisions—particularly in identifying early sucking problems that may signal neurological concerns requiring intervention [4,34].

When development was assessed at a corrected age of 12 months across the three groups, the mean PDI in the AI-based Dysfunction group was relatively low compared with the other groups; however, this difference was not statistically significant. This trend was observed in both the AI-classified and manually classified Dysfunction groups. These findings contrast with those of a previous study, which suggested that dysfunctional sucking patterns may predict later motor developmental challenges [23]. The discrepancy between our results and earlier research may be explained by limitations such as the small number of patients—particularly in the Dysfunction group—which reduced the statistical power to detect significant differences. Furthermore, although the AI model demonstrated high precision, its specificity was comparatively low, leading to a misclassification of clinical cases. Such misclassification may have contributed to inaccurate developmental predictions.

This study has some limitations. First, the external validity of our findings is limited by the choice of reference standard. Although VFSS is the gold standard for diagnosing dysphagia in infants, we used the Neonatal Oral Motor Assessment Scale (NOMAS) as a comparator due to ethical concerns related to radiation exposure and the practical constraints of performing VFSS in all preterm infants. While NOMAS is a widely used clinical alternative, its moderate validity and known inter-rater variability, particularly in disorganized sucking patterns, constrain the generalizability of our results to broader clinical settings. Inter-rater agreement between the two NOMAS-certified therapists yielded only a moderate weighted kappa (κ = 0.598), which implies that the manual classifications used as ground truths were not perfectly consistent. To partially address this concern, discordant cases were resolved through consensus discussion between the two raters; however, this procedure does not fully eliminate the variability inherent to subjective clinical judgment. Future studies should consider more reference standards. Second, improvement in peak detection is needed to prevent the misclassification of Normal cases as Disorganization, which often results from the incorrect splitting of continuous sucking within a segment. Third, tracking performance may be influenced by the quality of initial labels; therefore, the uncertainty associated with these labels should be minimized to ensure reliable analysis. Also, our AI-based analysis focused primarily on sucking patterns and did not account for structural abnormalities that are essential for accurately diagnosing Dysfunction cases. In addition, the AI-based analysis did not effectively detect resting periods, which were visually identified during manual assessment. This highlights the need for more comprehensive analytical approaches, including oral-facial structural assessment, to enhance diagnostic accuracy. Additionally, the diagnostic categorization logic applied in this study is rule-based and deterministic, with thresholds defined a prior in accordance with NOMAS clinical criteria. The AI component refers specifically to the deep learning-based key point tracking model (CoTracker2). No supervised training of the classification algorithm was performed. Accordingly, this study should be interpreted as a feasibility evaluation of the integrated framework rather than a validation of a trained AI classifier. Also, the analysis of neurodevelopmental outcomes at 12 months of corrected age was exploratory in nature and was underpowered, particularly given that only 2 infants were classified in the Dysfunction group. Cautious conclusions regarding predictive validity can be drawn from these data. A larger, prospectively powered study is required to determine whether AI-based feeding classification in the neonatal period is associated with later developmental outcomes. Finally, this study included only bottle-fed preterm infants from the outpatient clinic, which limited the number of children with swallowing dysfunction. Further validation is needed in children who are in NICU environments, or not fully orally fed, or in those who are breastfeeding to support the generalizability of the proposed framework.

## 5. Conclusions

This novel AI-based video analysis demonstrates preliminary potential as a noninvasive screening tool for non-trained people to evaluate swallowing function in preterm infants. However, further refinement and validation are warranted before clinical implementation. Given the misclassification rate and reliance on a subjective comparator, our findings should be interpreted as a proof-of-concept rather than a validated diagnostic tool.

## Figures and Tables

**Table 1:** Baseline characteristics of the study population (*n* = 58).

Variables	Values
Gestational age	
<28 weeks	20 (34.4)
28 ≤ GA < 32 weeks	25 (43.1)
32 ≤ GA < 37 weeks	13 (22.4)
Birth weight (g)	1189 ± 519
Corrected Age (at the time of participation, weeks)	5.1 ± 2.3
Sex (Male: Female)	38 (65.5): 20 (34.5)
Brain injury (*n*)	33 (56.8)
MDI of BSID-II at CA 12 months	86.0 ± 13.7
PDI of BSID-II at CA 12 months	78.1 ± 16.0

CA: Corrected age; values are mean ± SD or number (%).

**Table 2:** Comparison of NOMAS classification between AI-based analysis and manual expert evaluation.

Manual Classification	TP	FP	TN	FN	Sensitivity (%)	Specificity (%)	Accuracy (%)
Normal	16	1	32	9	64 [44.5–79.8]	96.97 [84.7–99.5]	82.76 [71.1–90.4]
Disorganization	30	9	18	1	96.8 [83.8–99.4]	66.67 [47.8–81.4]	82.76 [71.1–90.4]
Dysfunction	1	1	55	1	50 [9.5–90.5]	98.21 [90.6–99.7]	96.55 [88.3–99]

TP: True positive; FP: false positive; TN: true negative; FN: false negative; 95% confidence interval is indicated in [].

**Table 3:** Comparison of developmental indices according to NOMAS classification by manual and AI-based analyses.

	Manual Analysis						
Developmental Indices	Normal (*n* = 24)	Disorganization (*n* = 26)	Dysfunction (*n* = 2)	*p*-value ^†^	*p*-value ^††^	*p*-value ^†††^	*p*-value ^††††^
MDI	86.88 ± 12.00	86.19 ± 13.79	72.50 ± 33.23	0.853	0.615	0.655	0.364
PDI	77.13 ± 16.07	80.08 ± 15.66	63.00 ± 19.80	0.370	0.210	0.152	0.328
	**AI Analysis**						
Developmental Indices	Normal (*n* = 16)	Disorganization (*n* = 34)	Dysfunction (*n* = 2)	*p*-value ^†^	*p*-value ^††^	*p*-value ^†††^	*p*-value ^††††^
MDI	87.18 ± 12.55	85.18 ± 14.56	90.00 ± 8.49	0.546	0.944	0.604	0.818
PDI	78.31 ± 17.00	78.50 ± 15.94	68.50 ± 12.02	0.958	0.324	0.282	0.698

MDI: Mental Development Index; PDI: Psychomotor Development Index; AI, artificial intelligence; ^†^ *p* > 0.05 by Mann–Whitney. U test to compare Normal and Disorganization groups; ^††^ *p* > 0.05 by Mann–Whitney U test to compare Normal and Dysfunction groups; ^†††^ *p* > 0.05 by Mann–Whitney U test to compare Disorganization and Dysfunction groups; ^††††^ *p* > 0.05 by one way ANOVA to compare Normal, Disorganization, and Dysfunction groups.

## Data Availability

The datasets analyzed during the current study are available from the corresponding authors upon request.

## Supplementary Materials

The following supporting information can be downloaded at: https://www.mdpi.com/article/10.3390/children13040479/s1, This file contains Supplementary Materials, including representative examples of facial keypoint tracking (Figure S1) and the confusion matrix for the AI-based classification (Table S1), and the tracking video.

## Abbreviations

The following abbreviations are used in this manuscript:

BSID-II	Bayley Scales of Infant Development, Second Edition
CA	Corrected Age
FEES	Fiberoptic Endoscopic Evaluation of Swallowing
TP	True positive
FP	False positive
TN	True negative
FN	False negative
NOMAS	Neonatal Oral Motor Assessment Scale
NNS	Non-Nutritive Suck
MDI	Mental Development Index
PDI	Psychomotor Development Index
VFSS	Videofluoroscopic Swallowing Study
