# Supplementary material for: Artificial Intelligence-Based Video Analysis for Assessing Sucking Behavior in Preterm Infants: A Feasibility Study
Source: Children (Basel). 2026 Mar 30;13(4):479. doi: 10.3390/children13040479 (PMC13115115; doi:10.3390/children13040479)
Supplement: Supplementary file 1 [file children-13-00479-s001.zip › children-4167544-supplementary file.pdf]

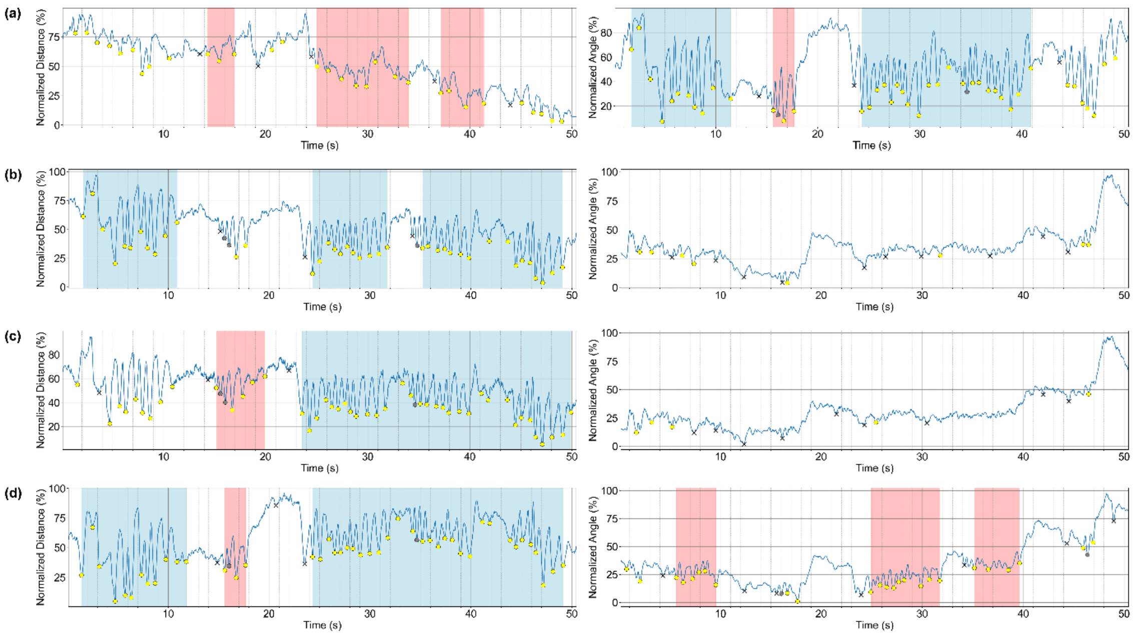

Figure S1. Representative examples of tracking facial keypoints: (A) eye–chin (B) glabella–chin (C) mandible–chin (D) mouth–chin.

Table S1. Confusion matrix of AI-based classification.

	Predicted: Normal	Predicted: Disorganization	Predicted: Dysfunction
Actual: Normal	16	8	1
Actual: Disorganization.	1	30	0
Actual: Dysfunction	0	1	1
