# Supplementary figures and images for: Artificial Intelligence-Based Video Analysis for Assessing Sucking Behavior in Preterm Infants: A Feasibility Study
Source: Children (Basel). 2026 Mar 30;13(4):479. doi: 10.3390/children13040479 (PMC13115115; doi:10.3390/children13040479)

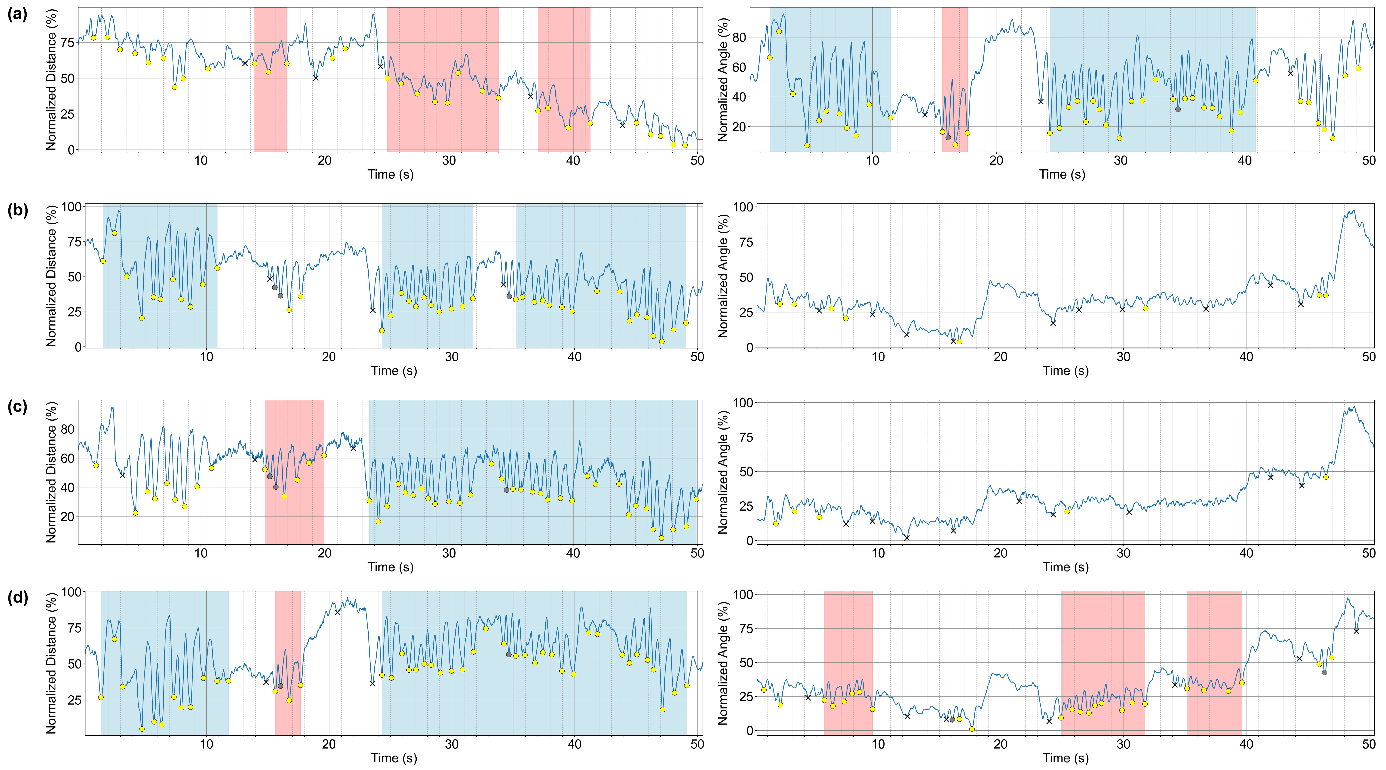

Supplement: Supplementary file 1 [file children-13-00479-s001.zip › Figure S1.png]
